# Morphology-engineered Ag/ZnO coral-like nanoplates for visible-light photocatalytic degradation of methyl orange and decolorization of palm oil mill effluent

**DOI:** 10.1039/d6ra06010b

**Published:** 2026-10-30

**Authors:** Piangjai Peerakiatkhajohn, Teera Butburee, Miaoqiang Lyu, Salwatee Yu-soh, Padsiriporn Budwang, Supphasin Thaweesak

**Affiliations:** a Faculty of Environment and Resource Studies, Mahidol University Nakhon Pathom 73170 Thailand piangjai.pee@mahidol.ac.th; b National Nanotechnology Center, National Science and Technology Development Agency 111 Thailand Science Park Pathum Thani 12120 Thailand teera.but@nanotec.or.th; c Nanomaterials Centre, School of Chemical Engineering and Australian Institute for Bioengineering and Nanotechnology (AIBN), The University of Queensland St Lucia QLD 4123 Australia m.lyu@uq.edu.au; d Department of Chemical Engineering, Faculty of Engineering, Burapha University Chon Buri 20131 Thailand supphasin@eng.buu.ac.th

## Abstract

Palm oil mill effluent (POME) is a complex industrial wastewater containing high organic loads and persistent colored constituents that are not completely removed by conventional biological treatment processes. In this study, porous coral-like ZnO nanoplates were synthesized *via* a urea-assisted hydrothermal method and Ag-modified ZnO was prepared by introducing silver nitrate into the precursor mixture prior to hydrothermal treatment to enhance visible-light photocatalytic activity. The effects of urea concentration and Ag loading on the morphology, surface characteristics, optical properties, and photocatalytic performance of the materials were systematically investigated. Among the pristine ZnO samples, ZnO-16U exhibited the highest photocatalytic activity, achieving approximately 90% methyl orange (MO) degradation under UV irradiation after 120 min despite having the lowest BET surface area, indicating that photocatalytic performance was governed by a combination of structural accessibility and optical/electronic properties. Ag incorporation markedly enhanced visible-light activity, with 5 wt% Ag/ZnO achieving approximately 92% MO degradation and 85% decolorization of post-treated POME after 120 min. For post-treated POME, 5 wt% Ag/ZnO also achieved 83% COD removal and 82% TOC removal, demonstrating substantial reduction of the residual organic load. The enhanced activity was associated with improved visible-light absorption through Ag-induced localized surface plasmon resonance and more efficient interfacial charge separation within the Ag/ZnO heterostructure. The 5 wt% Ag/ZnO catalyst retained approximately 79% POME decolorization after five reuse cycles, while very low Ag and Zn leaching indicated good material stability during repeated operation. The results demonstrate that combining morphology-engineered ZnO with Ag modification provides an effective strategy for developing visible-light-responsive photocatalysts for dye-contaminated water and post-treated POME from palm oil milling operations.

## Abstract 2

Palm oil mill effluent (POME) is a complex industrial wastewater containing high organic loads and persistent colored constituents that are not completely removed by conventional biological treatment processes.

## Graphical abstract

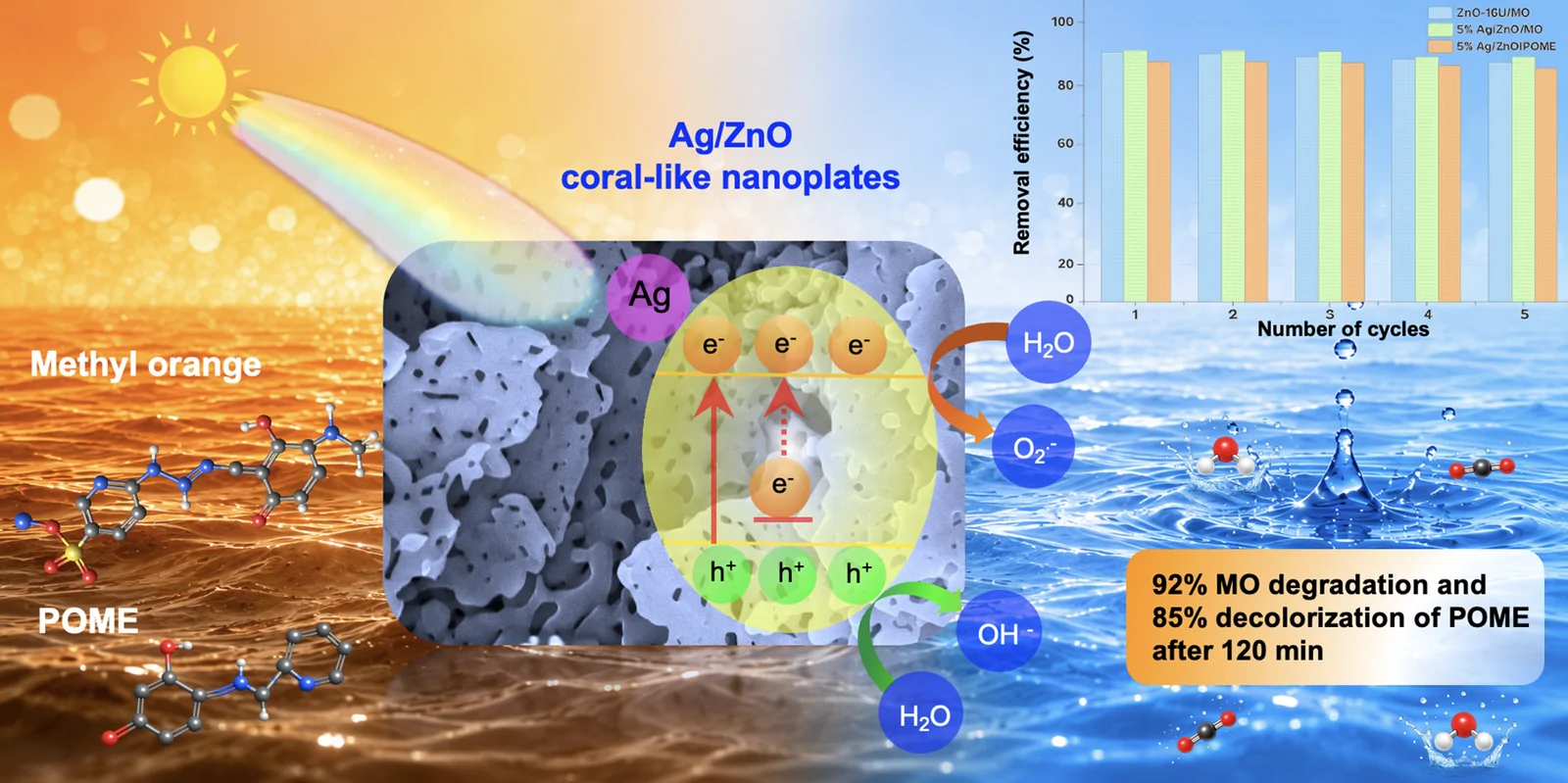

## 1 Introduction

The global consumption of vegetable oils has increased substantially over the past decade, rising from approximately 178 million tons in 2015 to more than 224 million tons in 2025.^1–3^ Among these, palm oil is the most widely produced, accounting for about one-third of global vegetable oil production.^4^ The dominance is due to its versatility in physicochemical properties and a diverse range of applications in the food industry, oleochemical and chemical industries, and the biofuel and energy sectors.^2^ The palm oil milling industry plays an important economic role and is mainly concentrated in tropical regions, particularly in Indonesia and Malaysia, which together account for over 80% of global production.^5,6^ Although the palm oil milling industry plays a vital role in supporting industrial growth and economic development, it continues to pose serious environmental challenges, primarily through the generation of solid waste, emissions of greenhouse gases and air pollutants, and the discharge of untreated wastewater. During palm oil production, approximately 10% of the fresh fruit bunch (FFB) is converted into oil, while the remaining 90% is generated as solid and liquid waste.^1,7^ Among these by-products, about 40% consist of biomass residues, such as empty fruit bunch (EFB), mesocarp fibers, and shells, whereas the remaining 60% is discharged as palm oil mill effluent (POME).^7–9^

POME is mainly generated from sludge separators, sterilizer condensate, and hydrocyclones.^10^ Fresh POME is a brownish, viscous liquid consisting of approximately 95–96% water, 0.6–0.7% residual oil, and 4–5% total solids. Its brownish coloration is attributed to the presence of organic compounds such as phenolics, pectin, tannins, lignin, and carotene.^11^ POME is recognized as a major environmental challenge because of its large wastewater generation volume, 2.5–3.0 tons of POME for every ton of crude palm oil produced,^10^ and extremely high organic loading, characterized by elevated concentrations of chemical oxygen demand (COD), biological oxygen demand (BOD), and total suspended solids (TSS).^10,12^ Biological treatment is widely employed for POME treatment due to its simple design, low energy demand, high treatment efficiency, and operational reliability.^9,11^ However, complete decolorization cannot be achieved using biological treatment processes alone.^13–15^

Advanced and hybrid treatment technologies such as advanced oxidation processes (AOPs), photocatalysis, membrane filtration, and integrated biological–chemical systems have attracted increasing attention for further removal of recalcitrant organic compounds, color, and residual pollutants in treated POME.^13,15,16^ Among these approaches, ZnO is particularly attractive because of its chemical stability and low toxicity.^17–19^ However, its practical photocatalytic performance is often restricted by rapid charge carrier recombination and limited visible-light utilization. Previous studies have primarily focused on tailoring ZnO morphology to enhance surface area and active sites,^18,20,21^ as well as incorporating noble metals such as Ag to improve light absorption and charge separation.^22,23^ While these strategies have demonstrated performance improvements, they have largely been investigated independently. Moreover, many Ag/ZnO systems have been evaluated using model pollutants under controlled laboratory conditions,^22–24^ whereas studies of morphology-engineered ZnO for POME treatment have typically relied on unmodified materials. Consequently, a comprehensive understanding of how morphology control and metal incorporation synergistically influence photocatalytic performance in complex wastewater systems remains limited.^16,25,26^ To further clarify the research gap and highlight the novelty of the present work, previously reported photocatalysts for methyl orange degradation and POME decolorization or treatment are summarized in Table 1. The comparison focuses on photocatalyst type, target pollutant or wastewater, irradiation condition, photocatalytic performance and key limitations of previous studies.

**Table 1:** Comparison of previously reported photocatalysts for methyl orange degradation and POME decolorization/treatment

Photocatalyst	Target pollutant or wastewater	Light source/condition	Key reported performance and limitations	Reference
Morphology-controlled ZnO and Ag–ZnO spherical flower-like structures	MO and RhB	UV-vis irradiation using Xe lamp	High MO and RhB degradation efficiency were reported. MO degradation occurred within 35 min. The study focused on model dyes only; POME was not investigated	27
ZnO–Ag polymer composite films	MO	Visible light	Efficient visible-light MO degradation was achieved. TOC analysis supported MO mineralization. The catalyst was immobilized in polymer films and was not tested with real wastewater	28
Ag-doped ZnO nanoparticles synthesized using *Cannabis sativa* extract	MO and Congo red	Solar light, pH 8	Ag–ZnO achieved approximately 94% MO degradation within 80 min. Bare ZnO achieved approximately 35% degradation. The study did not evaluate POME or industrial wastewater	29
Al-doped ZnO nanoparticles	MO	Visible-light irradiation	5 wt% Al/ZnO achieved approximately 99% MO degradation within 40 min. Metal modification enhanced visible-light activity. The study did not investigate Ag plasmonic modification or POME treatment	19
Co_3_O_4_–Ag photocatalyst	MO	UV and visible light	96.4% MO degradation was achieved after 30 h under UV light. 99.1% MO degradation was achieved after 6 h under visible light. The system was not ZnO-based and required relatively long irradiation time	30
Porous ZnO coral-like nanoplates	Organic dye	Simulated/visible-light-related irradiation	Porous coral-like ZnO showed high dye photodegradation and good regeneration. The study used unmodified ZnO. Ag modification and POME decolorization were not investigated	20
UV/ZnO system optimized by response surface methodology	POME	UV irradiation	Optimum COD removal of 55.30% was reported. The system was UV-driven and did not involve Ag modification	16
ZnO-PEG and ZnO–CC nanoparticles	Palm oil mill secondary effluent (POMSE)	UV irradiation	Color removal of 70.75% was reported. Turbidity reduction and COD removal reached 87.33% and 68.31%, respectively. Ag-induced visible-light enhancement was not investigated	25
Ag-doped TiO_2_	Recalcitrant POME	Visible-light irradiation	0.25 wt% Ag/TiO_2_ improved POME degradation to approximately 15.03%. Scavenging tests indicated the role of ˙OH radicals. The system was TiO_2_-based and showed lower POME degradation efficiency than the present work	31
Ag-titanate nanotube dual-layer hollow fiber membrane	Palm oil wastewater/aerobically treated POME (AT-POME)	Submerged membrane photoreactor	Ag-TNT membrane achieved 84.0% AT-POME color removal. The system combined photocatalysis and membrane filtration. The active phase was titanate-based, not Ag/ZnO	32

As shown in Table 1, previous ZnO and Ag/ZnO-based photocatalysts have demonstrated high degradation efficiencies toward model dye pollutants, including methyl orange. However, most of these studies were conducted using single-component dye solutions under controlled laboratory conditions, without evaluating catalyst performance in complex industrial wastewater matrices. In contrast, reported POME photocatalysis studies have mainly employed UV-activated ZnO, TiO_2_-based systems, Ag/TiO_2_ photocatalysts, or photocatalytic membrane processes. Therefore, the simultaneous integration of morphology-engineered ZnO, Ag-induced visible-light enhancement, methyl orange degradation, real POME decolorization, and catalyst reusability remains comparatively less explored.

To address this gap, the present study addresses this gap by developing a morphology-engineered Ag/ZnO photocatalyst system in which porous coral-like ZnO nanoplates were synthesized *via* a urea-assisted hydrothermal method, and Ag-modified ZnO nanoplates were prepared by introducing AgNO_3_ into the precursor mixture prior to the hydrothermal treatment step. The combined investigation of morphology engineering and Ag incorporation is necessary because these strategies influence different but complementary aspects of photocatalysis. Porous coral-like ZnO can improve surface accessibility, interconnected pore structure, and mass transport, whereas Ag nanoparticles enhance visible-light absorption through plasmonic effects and promote interfacial charge separation at the Ag/ZnO interface. By evaluating the developed materials using both MO as a model organic dye and post-treated POME obtained from an industrial source, this study provides a more realistic assessment of the catalytic applicability of Ag/ZnO for complex wastewater treatment.

## 2 Materials and methods

### 2.1 Materials and reagents

Urea (CH_4_N_2_O), zinc nitrate hexahydrate (Zn(NO_3_)_2_·6H_2_O), and silver nitrate (AgNO_3_) were purchased from Sigma-Aldrich (St. Louis, MO, USA), while ethanol (C_2_H_5_OH) was obtained from RCI Labscan. All chemical reagents used in this study were of analytical grade and used without further purification. Deionized water was employed for solution preparation and throughout all experiments. Post-treated POME was collected from the stabilization pond of the wastewater treatment pond system at a palm oil mill located in Chon Buri Province, Thailand. The post-treated POME was stored at 4 °C prior to use to minimize compositional changes before photocatalytic testing.

### 2.2 Synthesis of ZnO and Ag/ZnO nanostructures

Porous ZnO nanoplates were synthesized *via* a hydrothermal method using zinc nitrate hexahydrate as the precursor and urea as a structure-directing agent. The synthesis procedure was adapted with modifications from previous reports.^20,33^ In a typical synthesis, 4 mmol of Zn(NO_3_)_2_·6H_2_O was dissolved in 30 mL of deionized water to prepare the precursor solution. Separately, CH_4_N_2_O (8, 12, or 16 mmol) was dissolved in 20 mL of deionized water and used as the intermediate urea solution. The precursor solution was stirred for 15 min, followed by addition of the intermediate urea solution and the mixture was continuously stirred for 15 min to obtain a homogeneous turbid suspension with an approximate pH of 5. Subsequently, the resulting suspension was transferred into a 100 mL Teflon-lined stainless-steel autoclave and hydrothermally treated in a hot-air oven at 200 °C with a heating rate of 2 °C min^−1^ for 24 h. After the autoclave was naturally cooled to room temperature, the precipitate was collected, thoroughly rinsed with ethanol, and dried at 60 °C for 24 h. The dried product was then calcined in air at 600 °C for 2 h to obtain porous coral-like ZnO nanoplates. The obtained samples were denoted as ZnO-8U, ZnO-12U, and ZnO-16U, corresponding to the urea concentrations used during synthesis.

For the preparation of Ag-deposited ZnO-16U, AgNO_3_ was used as a source of silver and introduced into the precursor mixture at different amounts (1, 3, and 5 wt% relative to ZnO) under continuous stirring for 30 min prior to hydrothermal treatment. The resulting mixture was then subjected to the same hydrothermal conditions (200 °C for 24 h). Subsequently, the precipitate was collected, rinsed with ethanol, dried at 60 °C for 24 h, and calcined in air at 600 °C for 2 h. The resulting samples were denoted as 1 wt% Ag/ZnO, 3 wt% Ag/ZnO, and 5 wt% Ag/ZnO, corresponding to the nominal Ag loading used in the synthesis. A schematic illustration of the hydrothermal synthesis process for porous ZnO and Ag/ZnO coral-like nanoplates is presented in Fig. 1.

**Fig. 1:**
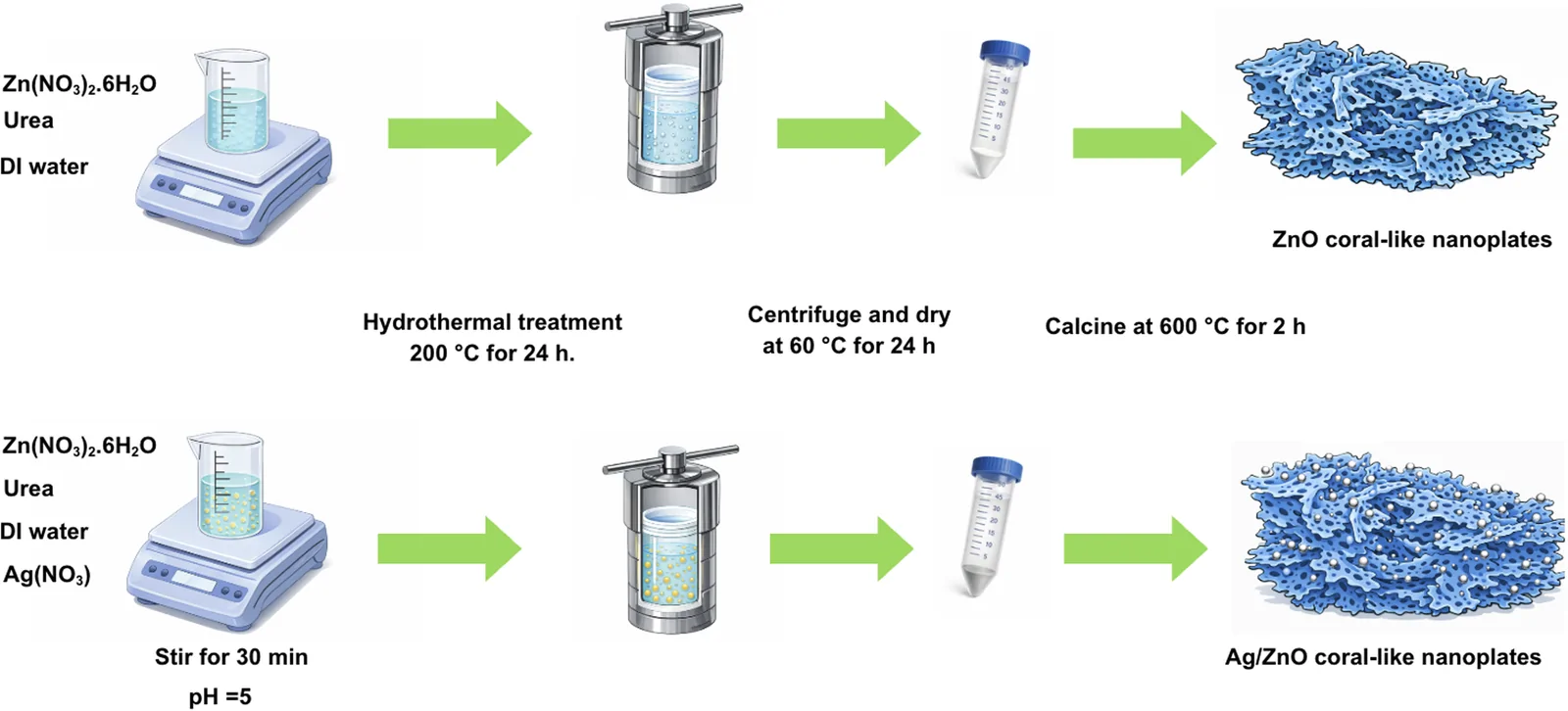
Schematic illustration of the hydrothermal synthesis process for porous ZnO and Ag/ZnO coral-like nanoplates.

### 2.3 Characterization

The physicochemical properties of the synthesized ZnO and Ag/ZnO samples were characterized using complementary analytical techniques. Surface morphology and microstructural features were examined using field-emission scanning electron microscopy (FE-SEM, JEOL JSM-IT500LA) operated at an accelerating voltage of 10 kV. Elemental composition and elemental distribution were analyzed using energy-dispersive X-ray spectroscopy (EDX) attached to the FE-SEM system at 15 kV. The morphology, crystallinity, and lattice structure of the samples were further investigated using transmission electron microscopy (TEM) and high-resolution TEM (HRTEM) on a JEOL JEM-2100 Plus microscope equipped with an energy-dispersive X-ray (EDX) detector and operated at 200 kV. The crystalline phases and structural purity of the samples were characterized by X-ray diffraction (XRD) using a BRUKER D2 PHASER with Cu Kα radiation (*λ* = 1.5406 Å) over a 2*θ* range of 20°–80°. The surface chemical states of Zn, O and Ag in Ag/ZnO was examined using X-ray photoelectron spectroscopy (XPS) analysis (Kratos Axis Supra Plus with monochromatic Al Kα X-ray). Nitrogen adsorption–desorption measurements were carried out to evaluate the textural properties of the samples. Prior to analysis, the samples were degassed under vacuum, and the specific surface area was calculated using the Brunauer–Emmett–Teller (BET) method, while the pore-size distribution and pore volume were determined using the Barrett–Joyner–Halenda (BJH) model. Surface functional groups and chemical interactions were analyzed using Fourier-transform infrared spectroscopy (FTIR, Thermo Fisher Scientific iS50) over the spectral range of 4000–650 cm^−1^. The optical absorption properties of the samples were investigated using a UV-vis spectrophotometer (Shimadzu UV-2600) in the wavelength range of 200–800 nm. The optical band-gap energies were estimated using the Tauc plot method based on the absorption spectra. Metal leaching from the photocatalysts after photocatalytic reactions was evaluated using inductively coupled plasma optical emission spectroscopy (ICP-OES, Agilent 5800 ICP-OES). Photoluminescence spectra (PL, HORIBA FluoroMax Plus) were recorded in the wavelength range of 350–600 nm at room temperature to evaluate the recombination behavior of photogenerated charge carriers.

### 2.4 Photocatalytic measurement

The photocatalytic performance of the synthesized ZnO and Ag/ZnO photocatalysts was evaluated using methyl orange as a model organic pollutant and post-treated palm oil mill effluent as a complex industrial wastewater matrix. MO (Sigma-Aldrich, St. Louis, MO, USA, 85% purity) was used without further purification, and the initial MO concentration was fixed at 50 mg L^−1^. The POME sample was collected from a stabilization pond of a palm oil mill treatment system and stored at 4 °C prior to use. The initial pH values of the MO solution and post-treated POME were approximately 6.5 and 7.0, respectively. No pH adjustment was performed before photocatalytic experiments in order to evaluate the catalyst performance under the original near-neutral conditions of the tested solutions.

In a typical experiment, 50 mg of photocatalyst was dispersed in 50 mL of MO solution or POME, corresponding to a catalyst dosage of 1.0 g L^−1^. The suspension was magnetically stirred for 30 min in the dark to establish adsorption–desorption equilibrium between the pollutant molecules and the photocatalyst surface. The concentration measured after this dark adsorption step was defined as C_0_ for kinetic analysis. The suspension was then irradiated under either UV-A or visible light depending on the experiment. UV-A irradiation was provided by four Philips TL-D UV-A fluorescent lamps with an emission maximum approximately at 365 nm, each with an electrical power of 18 W and a UV-A output of 3.9 W, giving a total UV-A output of 15.6 W. The irradiation area was 60 × 60 cm^2^. The distance between the lamp and the reactor was maintained at 10 cm. For visible-light experiments, irradiation was performed using an 18 W fluorescent lamp under the same reactor configuration. At fixed irradiation intervals of 30 min, aliquots of the suspension were withdrawn and immediately centrifuged at 4500 rpm for 10 min to separate the photocatalyst particles. The clear supernatant was analyzed using a UV-vis spectrophotometer. The residual MO concentration was determined from a calibration curve based on the absorbance at the maximum absorption wavelength of MO, approximately 464 nm.

The degradation efficiency was calculated as eqn (1):

1 Degradation (%) = [(*C*_0_ − *C*_*t*_)/*C*_0_] × 100

where *C*_0_ and *C*_*t*_ are the MO concentrations after dark adsorption and at irradiation time *t*, respectively. The apparent reaction rate constant was determined using the pseudo-first-order kinetic model shown in eqn (2):

2 ln(*C*_0_/*C*_*t*_) = *kt*

where *k* is the first-order rate constant, and *t* is the irradiation time.

Because POME is a complex multicomponent wastewater rather than a solution of a single defined compound, no concentration–absorbance calibration curve was applied. Accordingly, changes during photocatalytic treatment were monitored from the relative decrease in absorbance at 240 nm, which was used as an indicator of the reduction of UV-absorbing organic constituents. The decolorization efficiency was calculated as eqn (3):

3 Decolorization (%) = [(*A*_0_ − *A*_*t*_)/*A*_0_] × 100

where *A*_0_ and *A*_*t*_ are the POME absorbance values after dark adsorption and at irradiation time *t*, respectively. COD and TOC analyses were additionally performed to quantitatively evaluate the reduction of organic matter in post-treated POME. The apparent reaction rate constant was determined using the pseudo-first-order kinetic model shown in eqn (4):

4 ln(*A*_0_/*A*_*t*_) = *kt*

After photocatalytic treatment, the catalyst suspension was centrifuged, and the supernatant was filtered through a 0.22 µm membrane to remove residual catalyst particles. The filtrate was subjected to microwave-assisted acid digestion following US EPA Method 3015A with appropriate acid digestion conditions prior to analysis. The digested solutions were then analyzed for dissolved Ag and Zn to evaluate metal leaching from the Ag/ZnO catalyst after photocatalytic reaction.

## 3 Results and discussion

### 3.1 Morphology and BET surface area

The FE-SEM images presented in Fig. 2(a–f) reveal that the hydrothermally synthesized ZnO exhibits a complex, porous nanostructured coral-like architecture composed of irregularly shaped nanoplates.^21,33^ At low magnification, Fig. 2(a, c and e) the samples appear as loosely stacked assemblies of overlapping plate-like structures with irregular edges and abundant interparticle voids. At higher magnification, Fig. 2(b, d and f) the ZnO nanostructures display a highly perforated surface texture, characterized by numerous nanoscale pores distributed across the plate surfaces. These pores exhibit irregular shapes and sizes, typically in the range of approximately 10–30 nm, resulting in a sponge-like morphology. The lateral dimensions of the nanosheets are on the order of several hundred nanometers, although sizes vary due to aggregation and stacking effects. Such a porous nanoplates architecture is commonly associated with hydrothermal growth processes in which urea plays a critical role in regulating local pH and precipitation kinetics. The gradual decomposition of urea generates OH^−^ ions, promoting controlled nucleation and anisotropic crystal growth,^20,33,34^ while the release of NH_3_ can induce pore formation and structural restructuring during crystallization.^34,35^ As a result, a highly accessible porous framework is formed. A comparison of the three sample sets indicates an evolution in morphology with increasing urea concentration. The sample in Fig. 2(a and b) exhibits relatively compact stacking with smaller and less-defined pores and fine particulate features that may be attributed to secondary nucleation. Fig. 2(c and d) displays a more developed coral-like arrangement with increased pore definition. The sample in Fig. 2(e and f) demonstrates the most open and well-defined porous network, with larger void spaces and improved structural connectivity between nanoplates. This morphological evolution suggests that urea concentration strongly influences the growth dynamics, including nucleation rate, crystal orientation, and aggregation behavior, ultimately governing the formation of hierarchical porosity.^21,34,36,37^ Such a structure is advantageous for photocatalytic applications, as it provides a high density of accessible active sites, facilitates mass transport of reactants, and enhances light interaction within the porous network.^17,20,34,37^

**Fig. 2:**
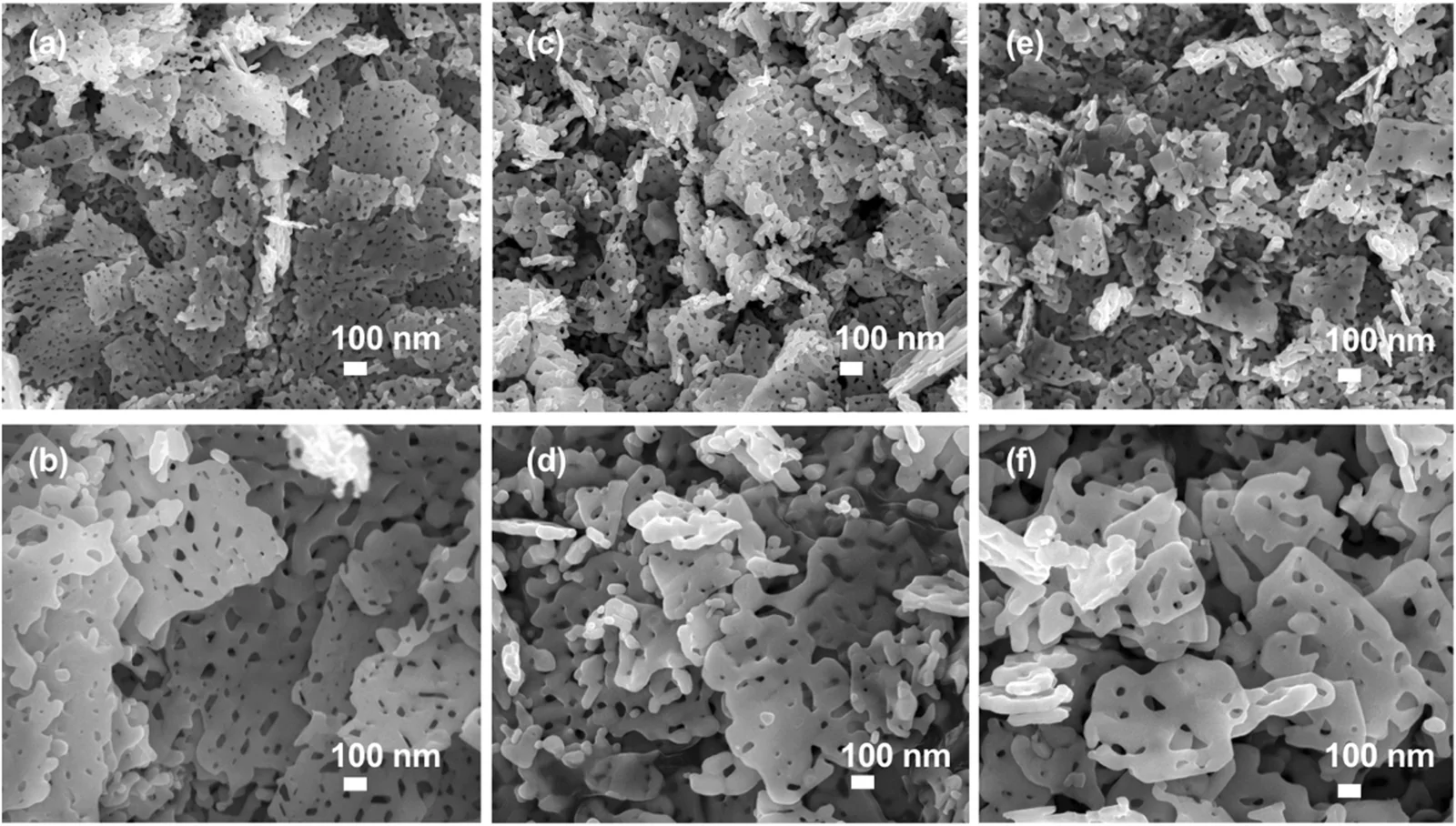
FE-SEM images of porous ZnO coral-like nanoplates synthesized with different urea concentrations: (a and b) 0.48 g, (c and d) 0.72 g, and (e and f) 0.96 g. Images (a, c and e) show low-magnification views, while (b, d and f) present high-magnification images.

The specific surface area and pore structure of the coral-like ZnO nanoplates were evaluated by N_2_ adsorption–desorption analysis. All samples exhibited type IV isotherms accompanied by H3 hysteresis loops, which are characteristic of mesoporous materials with slit-like pores formed by aggregated nanoplates.^18,20,33^ The specific surface area and pore volume were found to be strongly dependent on the urea concentration used during synthesis. The BET surface area decreased from 18.41 m^2^ g^−1^ for ZnO-8U to 13.03 m^2^ g^−1^ and 10.98 m^2^ g^−1^ for ZnO-12U and ZnO-16U, respectively. Similarly, the total pore volume decreased from 0.028 cm^3^ g^−1^ to 0.018 cm^3^ g^−1^ and 0.016 cm^3^ g^−1^. These results indicate a gradual reduction in accessible porosity with increasing urea concentration. The observed trend can be attributed to the role of urea in regulating the hydrothermal reaction environment. During thermal decomposition, urea gradually releases NH_3_ and CO_2_, resulting in a progressive increase in local alkalinity that influences nucleation and crystal growth processes. At higher urea concentrations, accelerated crystal growth and enhanced particle aggregation likely promote nanoplates stacking and structural densification, thereby reducing the available surface area and pore volume.^21,38^ The observed decrease in porosity is consistent with SEM observations, where samples synthesized with higher urea concentrations exhibited greater stacking and reduced structural openness. From a functional perspective, although a highly porous structure is beneficial for adsorption and mass transport, excessive densification may limit reactant accessibility and diffusion within the catalyst framework. Therefore, optimizing the balance between structural openness and crystallinity is critical for achieving improved photocatalytic performance.^18,37,39^ Thus, although increasing urea concentration reduces the overall surface area, it simultaneously promotes structural features that may favor photocatalytic reactions through enhanced electronic and optical properties.

### 3.2 Elemental composition, chemical states, and optical properties

Fig. 3(a–c) presents FE-SEM images of ZnO structures modified with different Ag loadings (1, 3, and 5 wt%). The results show that all Ag/ZnO samples retained the characteristic coral-like nanoplate architecture composed of interconnected and perforated nanoplates assembled into porous aggregates. This observation indicates that Ag incorporation did not significantly disrupt the hydrothermally formed ZnO nanostructure.^24,40^ At low Ag loading (1 wt% Ag; Fig. 3(a)), the ZnO porous nanoplates exhibited relatively smooth surfaces with only a limited number of bright nanoscale features, which can be attributed to dispersed Ag nanoparticles. As the Ag loading increased to 3 wt% (Fig. 3(b)), the surface became rougher and more densely decorated, suggesting increased surface coverage by Ag-containing nanoparticles. At 5 wt% Ag loading Fig. 3(c), the nanoplate surface appears more densely decorated, with locally aggregated regions indicating the onset of particle coalescence. Despite the increased Ag deposition, the porous nanoplates morphology remains largely preserved, confirming that the modification process does not disrupt the structural integrity of ZnO. The EDX spectrum and elemental mapping of the 5 wt% Ag/ZnO sample, shown in Fig. 3(d), confirm the presence of Zn, O, and Ag in the composite. Zn and O were distributed throughout the nanoplate structure, while Ag was detected across the ZnO surface with some localized Ag-rich regions. These results indicate that Ag species were successfully introduced and distributed on the ZnO nanoplates. However, excessive Ag loading may lead to partial pore blockage and the formation of recombination centers, depending on the degree of nanoparticle aggregation.^24,27^ TEM analysis of the 5 wt% Ag/ZnO sample, shown in Fig. 3(e), provides further morphological evidence of Ag deposition on the ZnO nanoplates. Darker contrast regions were observed on the ZnO framework, which can be attributed to Ag nanoparticles because of the higher electron density of Ag compared with ZnO.^22,40^ HRTEM analysis, shown in Fig. 3(f), further supports the coexistence of crystalline ZnO and Ag. The lattice fringe spacing of approximately 0.264 nm can be assigned to the (002) plane of wurtzite ZnO, while the spacing of approximately 0.241 nm corresponds to the (111) plane of face-centered cubic Ag.^41–43^ These observations support the formation of Ag-decorated ZnO heterostructures. The intimate contact between Ag nanoparticles and ZnO nanoplates is expected to facilitate interfacial charge transfer, which is beneficial for suppressing electron–hole recombination during photocatalysis.^22–24^

**Fig. 3:**
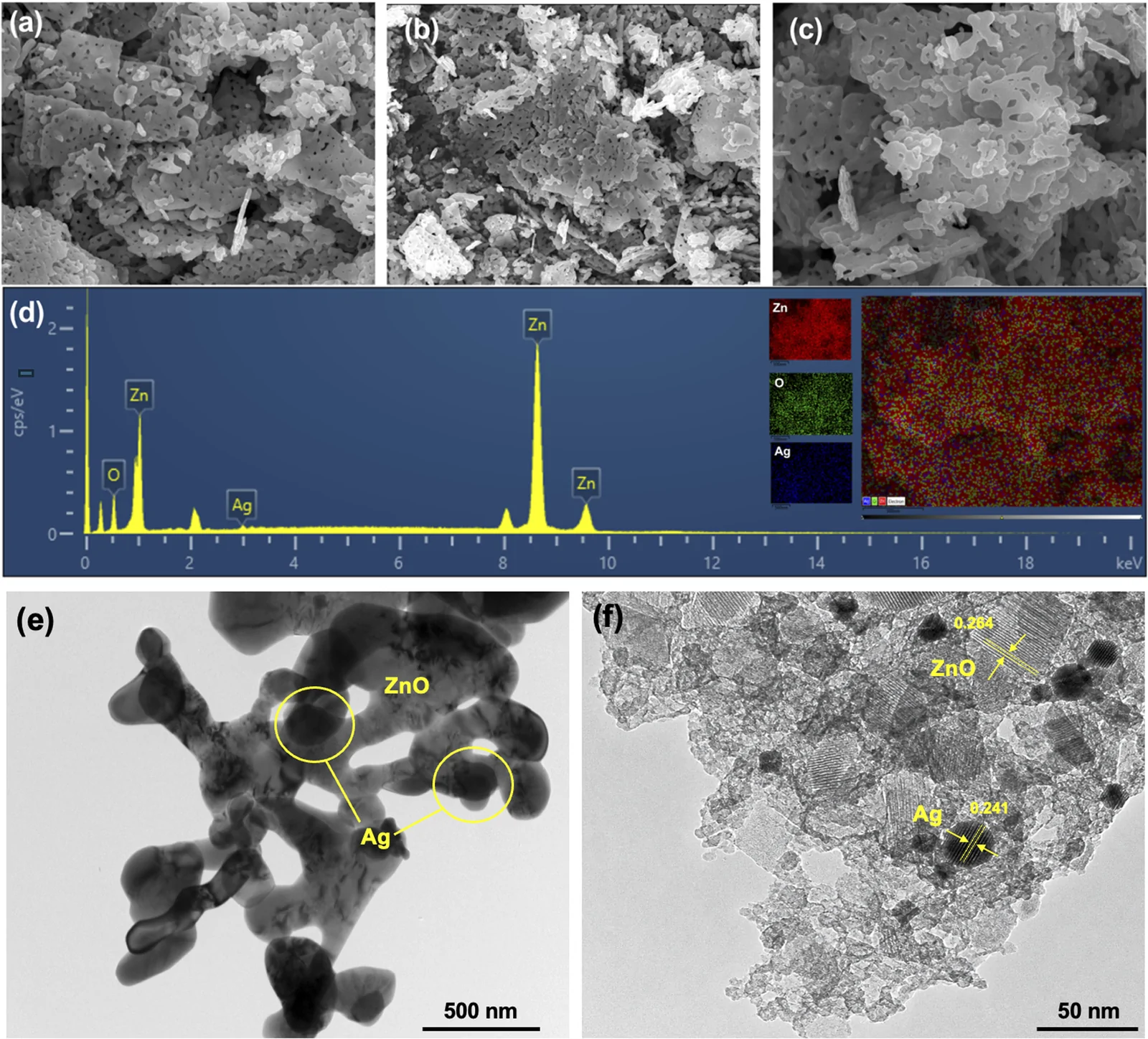
FE-SEM images of Ag-modified ZnO coral-like nanoplates synthesized with different Ag loadings: (a) 1 wt%, (b) 3 wt%, and (c) 5 wt%; (d) EDS spectrum and corresponding elemental mapping of the 5 wt% Ag/ZnO sample. (e) TEM image, and (f) HRTEM image of 5 wt% Ag/ZnO sample.

Fig. 4(a) shows the XRD patterns of pristine ZnO samples (ZnO-8U, ZnO-12U, and ZnO-16U) and Ag-modified ZnO-16U coral-like nanoplates (1, 3, and 5 wt% Ag/ZnO). All ZnO samples exhibited diffraction peaks that correspond to the hexagonal wurtzite structure (JCPDS no. 01-080-0075), confirming the successful formation of the crystalline ZnO structure.^44,45^ After Ag incorporation, the characteristic diffraction peaks of ZnO remained clearly observable, indicating that the crystal structure of ZnO was preserved during Ag modification. In addition, weak diffraction peaks located at approximately 38.1°, 44.3°, 64.4°, and 77.4° were detected, corresponding to the (111), (200), (220), and (311) planes of face-centered cubic Ag (JCPDS no. 01-087-0597).^22,23,44^ This result confirms the presence of crystalline Ag in the modified samples. No additional impurity peaks were detected within the detection limit of XRD, suggesting that the Ag modification process did not generate secondary crystalline phases other than ZnO and Ag. The relatively weak Ag diffraction peaks are consistent with the low Ag loading and the formation of Ag-containing nanodomains on the ZnO surface.^24,40^

**Fig. 4:**
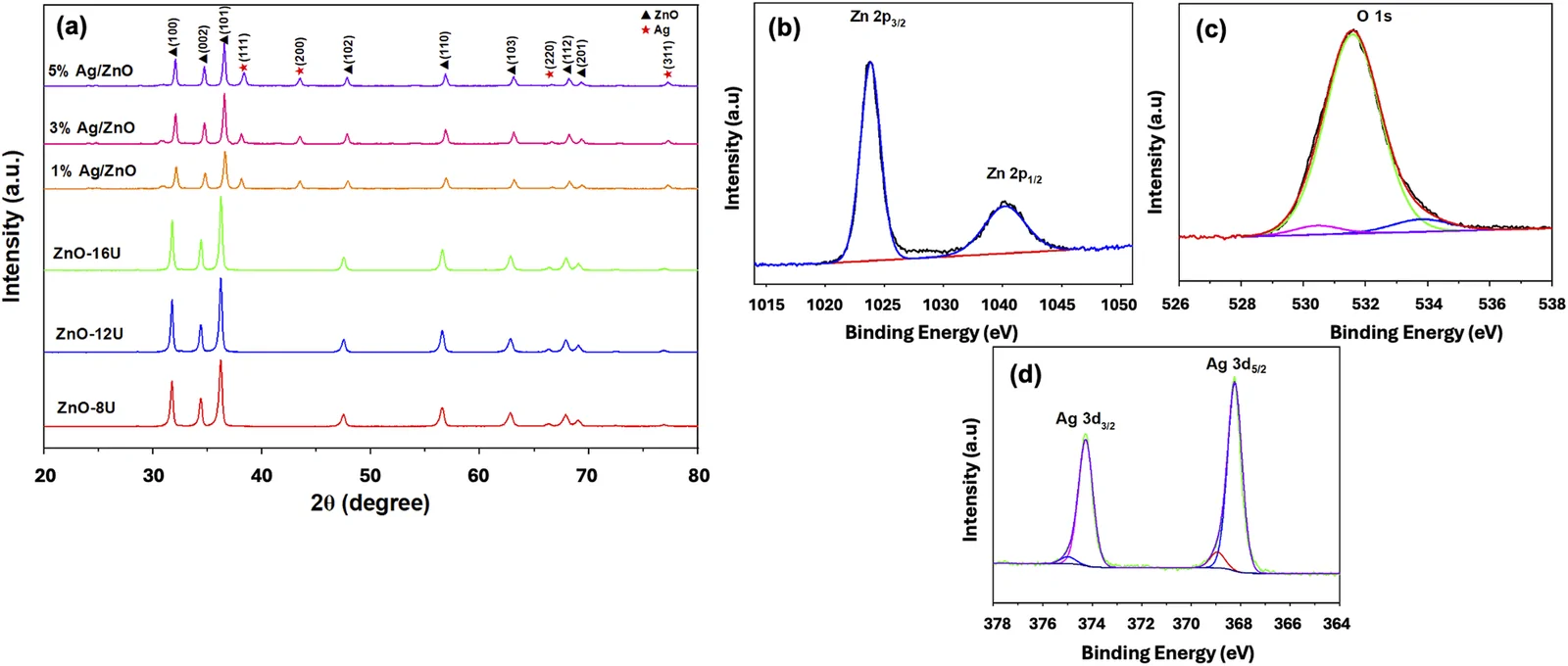
(a) XRD patterns of pristine ZnO samples (ZnO-8U, ZnO-12U, and ZnO-16U) and Ag-modified ZnO-16U samples with different Ag loadings of 1, 3, and 5 wt%. High-resolution XPS spectra of the 5 wt% Ag/ZnO sample: (b) Zn 2p, (c) O 1s, and (d) Ag 3d.

XPS analysis was further performed to investigate the surface chemical states of the 5 wt% Ag/ZnO sample. As shown in Fig. 4(b), the high-resolution Zn 2p spectrum exhibited two characteristic peaks at 1021 eV and 1040 eV corresponding to Zn 2p_3/2_ and Zn 2p_1/2_, respectively,^46^ confirming the presence of Zn species in the ZnO framework.^24,29^ The retention of these Zn 2p features indicates that the ZnO lattice was maintained after Ag modification, consistent with the XRD results.^22,46^ The O 1s spectrum shown in Fig. 4(c) was deconvoluted into three oxygen-related components. The low-binding-energy component at approximately 530 eV can be assigned to lattice oxygen in Zn–O bonds, while the intermediate component at approximately 532 eV is associated with defect-related oxygen species, which may be related to oxygen-deficient regions and/or surface hydroxyl groups. The high-binding-energy component can be attributed to adsorbed oxygen-containing species, such as surface hydroxyl groups, adsorbed water, or carbonate-related species.^46–48^ The presence of the defect-related O 1s component supports the existence of defect-associated surface oxygen species in the Ag/ZnO sample. These sites may contribute to enhanced adsorption and activation of oxygen molecules, thereby promoting the formation of reactive oxygen species during photocatalysis.^46,48^ The Ag 3d spectrum in Fig. 4(d) exhibited two characteristic peaks at 366.51 eV and 372.51 eV corresponding to Ag 3d_5/2_ and Ag 3d_3/2_, respectively, confirming the presence of Ag species on the ZnO surface.^23,46,47^ This result is consistent with the EDX, XRD, TEM and HRTEM observations, which collectively support the formation of Ag-decorated ZnO heterostructures.^22,24^ Therefore, the XRD and XPS results provide complementary evidence that the Ag modification preserves the ZnO framework while introducing Ag species and surface oxygen-related defect sites that contribute to enhanced visible-light photocatalytic performance.^22,23,46^

The FTIR spectra of ZnO nanoplates synthesized with different urea concentrations are presented in Fig. 5(a). All samples exhibited a broad absorption band in the range of 3200–3600 cm^−1^, which is associated with O–H stretching vibrations of surface hydroxyl groups and adsorbed water molecules.^18,37^ A characteristic absorption band observed below 600 cm^−1^ corresponds to Zn–O lattice vibration, confirming the formation of ZnO in all samples.^49^ The observation of this feature in all spectra indicates that the crystal framework of ZnO remains intact regardless of the urea concentration used during synthesis. In addition, weak absorption bands are detected in the mid-infrared region. Features around 2850–2950 cm^−1^ and 1000–1500 cm^−1^ were detected and can be attributed to C–H stretching vibrations, and C–O, C–O–C vibrations, respectively, suggesting the presence of residual organic species or carbonate groups on the surface. The relative intensities of the hydroxyl and carbon-related bands varied slightly among the samples, ZnO-8U, ZnO-12U, and ZnO-16U, indicating that the urea concentration influences surface chemistry, particularly the degree of hydroxylation and residual species adsorption. These surface functionalities are known to play an important role in photocatalytic reactions by facilitating pollutant adsorption and the generation of reactive oxygen species. The FTIR spectra of ZnO nanoplates incorporated with Ag at different loadings are presented in Fig. 5(b). The incorporation of Ag does not significantly modify the Zn–O lattice vibrations, indicating that the ZnO crystal framework remains intact. However, a slight enhancement and additional feature in the 500–600 cm^−1^ region was observed, which can be attributed to Ag–O vibrations associated with surface Ag–O species, partially oxidized silver, or interfacial Ag–O–Zn bonding.^23,24,40^ The O–H band in the 3200–3600 cm^−1^ region remains prominent after Ag loading, suggesting that surface hydroxyl groups and adsorbed water molecules are still present on the catalyst surface. This is beneficial for photocatalytic applications, as surface hydroxyl groups contribute to the formation of reactive hydroxyl radicals (˙OH) under irradiation. The FTIR results confirm formation of ZnO and Ag incorporation occurs primarily at the surface without disrupting the ZnO lattice, enabling the formation of a functional heterostructure. The UV-vis absorption spectra of ZnO nanoplates synthesized with different urea concentrations are shown in Fig. 5(c). All samples exhibited strong absorption in the ultraviolet region with an absorption edge located at approximately 375–390 nm, corresponding to the intrinsic band-edge transition of ZnO, which is associated with its wide band gap (*E*_g_ ≈ 3.2 eV) and characteristics of crystalline ZnO.^17,18,50^ The similarity of the absorption edges for ZnO-8U and ZnO-12U suggests that urea concentration has limited effect on the intrinsic band structure of ZnO. In contrast, ZnO-16U displayed a noticeable absorption tail extending into the visible region beyond 400 nm. Such extended absorption is tentatively attributed to defect-induced sub-band-gap states in ZnO, which may arise from oxygen-deficient sites, zinc interstitials, and/or other structural defects generated during urea-assisted hydrothermal synthesis. These defect-related states can introduce localized energy levels within the band gap and enable sub-band-gap optical transitions, thereby enhancing visible-light absorption.^17,18,37^

**Fig. 5:**
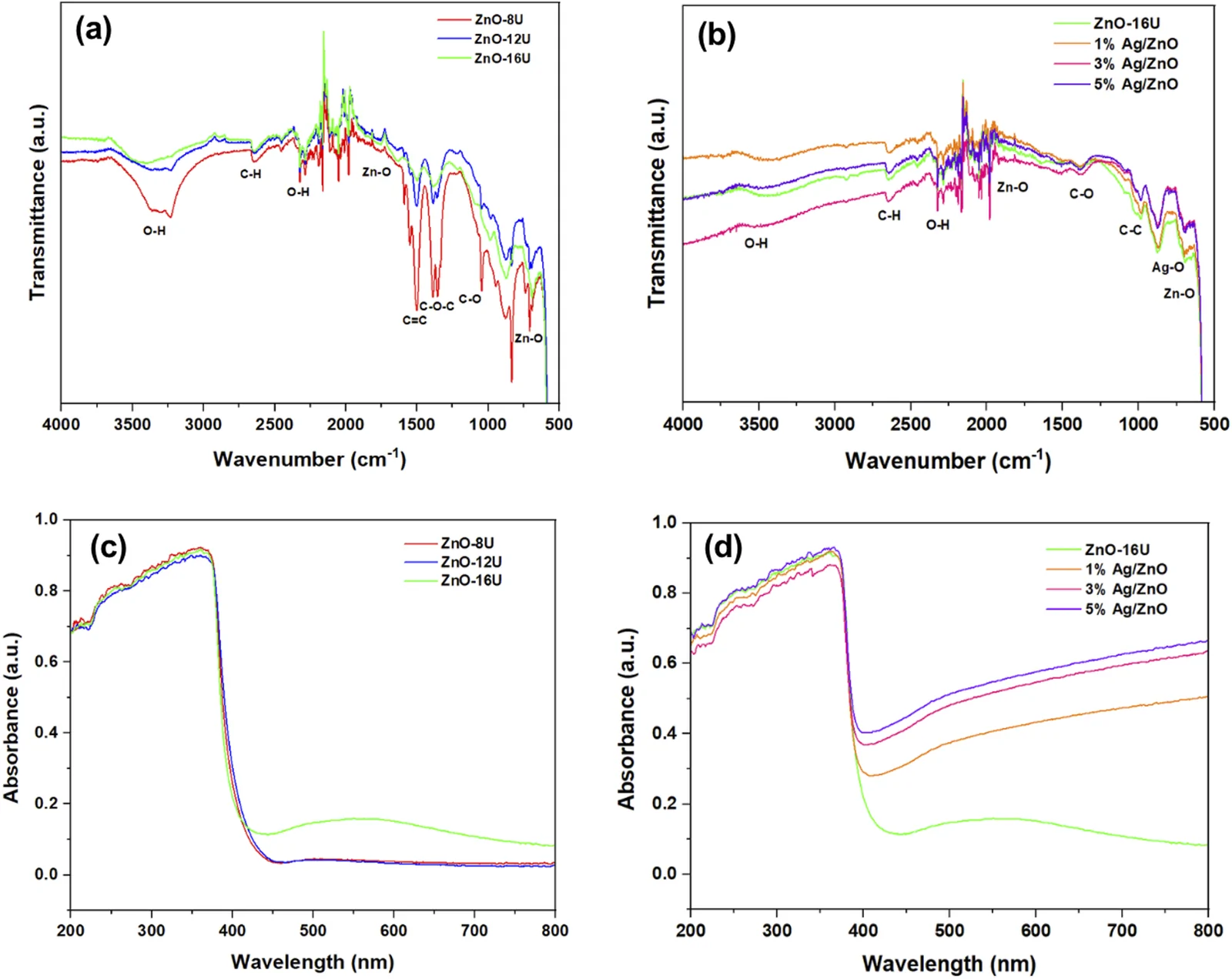
(a) FTIR spectra of ZnO nanoplates, (b) FTIR spectra of ZnO-16U and Ag-modified ZnO-16U samples, (c) UV-vis absorption spectra of ZnO nanoplates, and (d) UV-vis absorption spectra of ZnO-16U and Ag-modified ZnO-16U samples.

A significant enhancement in visible-light absorption was observed for Ag-modified ZnO samples, as shown in Fig. 5(d). The absorption intensity across the visible region (approximately 400–800 nm) increased as the Ag loading increased (5 wt% > 3 wt% > 1 wt%). This behavior is characteristic of plasmonic metal–semiconductor systems and is attributed to the localized surface plasmon resonance (LSPR) of Ag nanoparticles, which extends the optical response of ZnO into the visible region.^23,24,27^ The plasmonic excitation of Ag can generate energetic electrons, which may be transferred to the conduction band of ZnO or participate in the reduction of dissolved oxygen to form superoxide radicals (˙O_2_^−^), while holes in the valence band oxidize surface hydroxide ions or water molecules to form hydroxyl radicals (˙OH).^51–53^ These reactive species are highly oxidative and play a crucial role in the degradation and mineralization of organic pollutants into CO_2_ and H_2_O. In addition, the Ag/ZnO interface forms a Schottky-type junction, where Ag can act as an electron-accepting site to trap photogenerated electrons and suppress electron–hole recombination. Therefore, Ag plays a dual role as a plasmonic light absorber and an interfacial charge-transfer mediator.^22–24,40^ Photoluminescence spectroscopy (PL) was further used to evaluate the recombination behavior of photogenerated charge carriers, and the spectra are provided in Fig. S1. The pristine ZnO samples exhibited emission bands near 390–400 nm and a broad visible emission around 500–550 nm, which can be associated with near-band-edge recombination and defect-related radiative recombination, respectively.^24,46^ Among the pristine ZnO samples, ZnO-16U showed lower PL intensity than ZnO-8U and ZnO-12U, suggesting reduced radiative recombination of photogenerated charge carriers. After Ag modification, the PL intensity decreased markedly, with 5 wt% Ag/ZnO showing the lowest emission intensity. This result suggests that Ag incorporation suppresses electron–hole recombination and facilitates interfacial charge separation,^46,54^ which is consistent with the improved visible-light photocatalytic activity of Ag/ZnO.

### 3.3 Photocatalytic degradation of model dye and real wastewater over ZnO and Ag/ZnO nanostructures

Preliminary experiments were conducted to evaluate the effects of key operating parameters on MO degradation using ZnO-16U under UV-light irradiation, including the initial MO concentration, photocatalyst dosage, and reaction volume under a fixed catalyst mass. These parameters were examined because dye concentration and catalyst loading are known to strongly affect heterogeneous photocatalytic degradation through changes in photon penetration, pollutant adsorption, active-site availability, and catalyst dispersion.^55,56^ The results are provided in Fig. S2. The degradation efficiency decreased with increasing initial MO concentration, which can be attributed to greater competition of dye molecules for available active sites and reduced light penetration at higher dye concentrations.^55^ The catalyst dosage study showed that 1.0 g L^−1^ provided the highest degradation efficiency, whereas excessive catalyst loading reduced the activity, probably because of increased light scattering, shielding, and particle aggregation.^28,55^ In addition, varying the reaction volume under a fixed catalyst mass showed that smaller solution volumes enhanced the apparent degradation efficiency because of the higher catalyst/solution and catalyst/dye ratios. Based on these preliminary results, 50 mg of photocatalyst dispersed in 50 mL of MO solution, corresponding to a catalyst dosage of 1.0 g L^−1^, was selected for the subsequent photocatalytic activity tests. The photocatalytic performance of the synthesized ZnO and Ag/ZnO coral-like nanoplates was then systematically evaluated using methyl orange (MO) as a model organic pollutant, followed by testing with post-treated palm oil mill effluent (POME). The use of a model dye allows an assessment of intrinsic photocatalytic activity, while real wastewater evaluation provides insight into the catalyst performance in a multicomponent matrix containing various organic compounds and verifies the applicability of the catalyst under more complex wastewater conditions. Fig. 6(a–c) present the photocatalytic degradation behavior of MO over ZnO catalysts synthesized with different urea concentrations (ZnO-8U, ZnO-12U, and ZnO-16U). As shown in Fig. 6(a), a decrease in MO concentration was observed during the initial dark stage, indicating adsorption–desorption equilibrium between the dye molecules and catalyst surface.^17,20^ Upon UV irradiation, all ZnO samples exhibited a continuous reduction in MO concentration. The degradation efficiency increased with increasing urea content during synthesis, reaching approximately 78%, 85%, and 90% after 120 min for ZnO-8U, ZnO-12U, and ZnO-16U, respectively. The enhanced performance of ZnO-16U can be attributed to the structural and optical characteristics developed during urea-assisted hydrothermal synthesis. Although ZnO-16U exhibits a lower BET surface area than ZnO-8U and ZnO-12 U, FE-SEM analysis reveals a more interconnected porous structure, while UV-vis spectroscopy indicates enhanced visible-light absorption associated with defect-related electronic states. This observation suggests that photocatalytic performance is not governed solely by surface area or pore volume. Instead, factors such as structural accessibility, defect-related electronic states, charge transport characteristics, and light-harvesting capability appear to play a more significant role in determining photocatalytic efficiency.^17,18,37,50^ The kinetic plots in Fig. 6(b) show a good linear relationship between ln(*C*_0_/*C*_*t*_) and irradiation time, indicating that MO degradation can be described by a pseudo-first-order kinetics.^20,57^

**Fig. 6:**
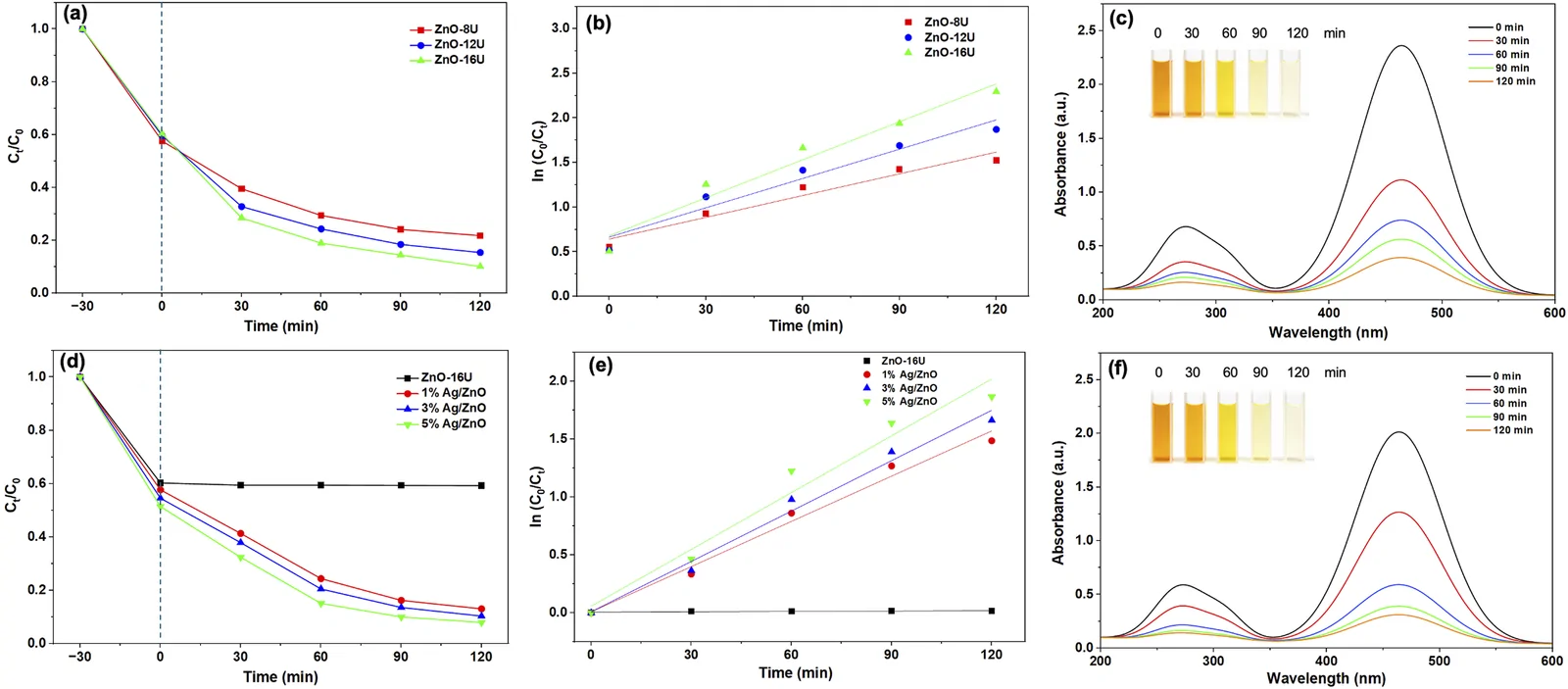
Photocatalytic degradation of MO over ZnO and Ag/ZnO coral-like nanoplates. (a) MO degradation profiles under UV irradiation over ZnO-8U, ZnO-12U, and ZnO-16U, (b) corresponding pseudo-first-order kinetics plots, and (c) UV-vis spectral evolution of MO over ZnO-16U, (d) MO degradation profiles under visible-light irradiation over ZnO-16U and Ag-modified ZnO-16U samples with different Ag loadings of 1, 3, and 5 wt%, (e) corresponding pseudo-first-order kinetics plots, and (f) UV-vis spectral evolution of MO over 5 wt% Ag/ZnO. Insets show the corresponding color changes during photocatalytic treatment.

The calculated rate constants (*k*) increase from 0.0091, 0.0126, and 0.0161 min^−1^ for ZnO-8U, ZnO-12U, and ZnO-16U, respectively, confirming the enhanced reaction kinetics of ZnO-16U. The evolution of the UV-vis absorption spectra during the photocatalytic degradation process, shown in Fig. 6(c), provides further support for the degradation process. The characteristic absorption band of MO at approximately 460–480 nm gradually decreases with irradiation time, indicating progressive destruction of the azo chromophore.^57,58^ The absence of significant peak shifts suggests that degradation proceeds predominantly through chromophore breakdown. The corresponding color change observed in the inset photographs further confirms the effective degradation of the dye solution. Based on the photocatalytic performance results, ZnO-16U was selected as the optimal catalyst for further modification with Ag nanoparticles to enhance activity under visible light irradiation. Fig. 6(d–f) present the visible light photocatalytic degradation of MO over ZnO-16U and Ag-modified ZnO-16U catalysts. As shown in Fig. 6(d), pristine ZnO-16U exhibits limited photocatalytic activity under visible light, due to its wide band gap and restricted absorption in the visible region. In contrast, all Ag-modified samples showed significantly enhanced degradation performance,^22–24^ with degradation efficiencies increasing to approximately 85%, 89%, and 92% for 1 wt%, 3 wt%, and 5 wt% Ag/ZnO, respectively. The corresponding pseudo-first-order rate constants in Fig. 6(e) increased from 0.0131 to 0.0146 and 0.0171 min^−1^ with increasing Ag loading. The enhanced performance is consistent with the structural and optical characterization results. UV-vis analysis demonstrates progressively stronger visible-light absorption with increasing Ag content, while XRD, EDX, TEM, and HRTEM confirm the successful formation of Ag/ZnO heterostructures. The improved photocatalytic activity can be attributed to the dual role of Ag as a plasmonic light absorber and an interfacial charge-transfer mediator.^22–24,40^ The gradual decrease of the MO absorption band shown in Fig. 6(f) further confirms the accelerated degradation process. Within the investigated Ag loading range, photocatalytic activity increases progressively with increasing Ag loading, reaching a maximum at 5 wt% Ag/ZnO. This result indicates that the beneficial effects of enhanced visible-light absorption and improved charge separation outweigh potential negative effects such as surface coverage or light scattering that may occur at higher metal loading levels.

To assess the performance of the synthesized photocatalysts in a more complex wastewater matrix, photocatalytic experiments were subsequently conducted using post-treated POME, which contains a heterogeneous mixture of organic compounds and colored constituents.^13,16,25,26^Fig. 7(a–c) presents the photocatalytic decolorization behavior of ZnO-8U, ZnO-12U, and ZnO-16U under UV irradiation. As shown in Fig. 7(a), all three ZnO samples exhibited a continuous decrease in *A*/*A*_0_ with irradiation time, indicating progressive reduction of UV-absorbing constituents in the post-treated POME. After 120 min of irradiation, ZnO-16U achieved the highest decolorization efficiency of approximately 80%, followed by ZnO-12U and ZnO-8U at approximately 76% and 71%, respectively. This activity order is consistent with the trend observed for MO degradation, confirming that ZnO-16U also exhibits the best photocatalytic performance in the more complex POME matrix. The corresponding kinetic plots in Fig. 7(b) further confirm the superior activity of ZnO-16U. This result suggests that the more open coral-like porous architecture, improved structural connectivity, and defect-related optical properties of ZnO-16U are beneficial for POME decolorization, despite its lower BET surface area. The UV-vis spectral evolution of POME over ZnO-16U, shown in Fig. 7(c), displays a gradual decrease in the broad absorption profile across the UV-visible region during irradiation. The inset photographs show a clear color change from dark brown to pale yellow, confirming the progressive decolorization of POME under UV irradiation. After confirming ZnO-16U as the most effective pristine ZnO catalyst for POME decolorization, Ag-modified ZnO catalysts were evaluated under visible-light irradiation. As shown in Fig. 7(d), Ag-modified catalysts consistently outperformed pristine ZnO-16U, achieving decolorization efficiencies of approximately 75%, 80%, and 85% for 1 wt% Ag/ZnO, 3 wt% Ag/ZnO, and 5 wt% Ag/ZnO, respectively, after 120 min. The corresponding rate constants in Fig. 7(e) increased from 0.002 min^−1^ for ZnO-16U to 0.0081 min^−1^, 0.0095 min^−1^, and 0.0107 min^−1^ for 1 wt% Ag/ZnO, 3 wt% Ag/ZnO, and 5 wt% Ag/ZnO, respectively. Although the POME decolorization efficiencies were lower than those observed for MO, the activity trend remained consistent. The lower reaction rates can be attributed to the complex wastewater matrix, in which organic species, suspended matter, and colored constituents may compete for active sites and attenuate incident light. The UV–Vis spectra in Fig. 7(f) show a gradual decrease in the broad absorption profile of POME throughout irradiation, indicating progressive reduction of colored organic constituents. Unlike the model dye system, no distinct absorption peak is observed because POME contains a heterogeneous mixture of organic compounds.^16,25,26^ The inset image further confirms the visible decolorization process under visible-light irradiation. These results demonstrate that the combination of morphology engineering and Ag incorporation significantly enhances photocatalytic performance under visible-light irradiation. The superior activity of Ag/ZnO, particularly the 5 wt% Ag/ZnO sample, highlights the importance of simultaneously optimizing structural accessibility, optical absorption, and interfacial charge-transfer processes for the treatment of both model pollutants and industrial wastewater.

**Fig. 7:**
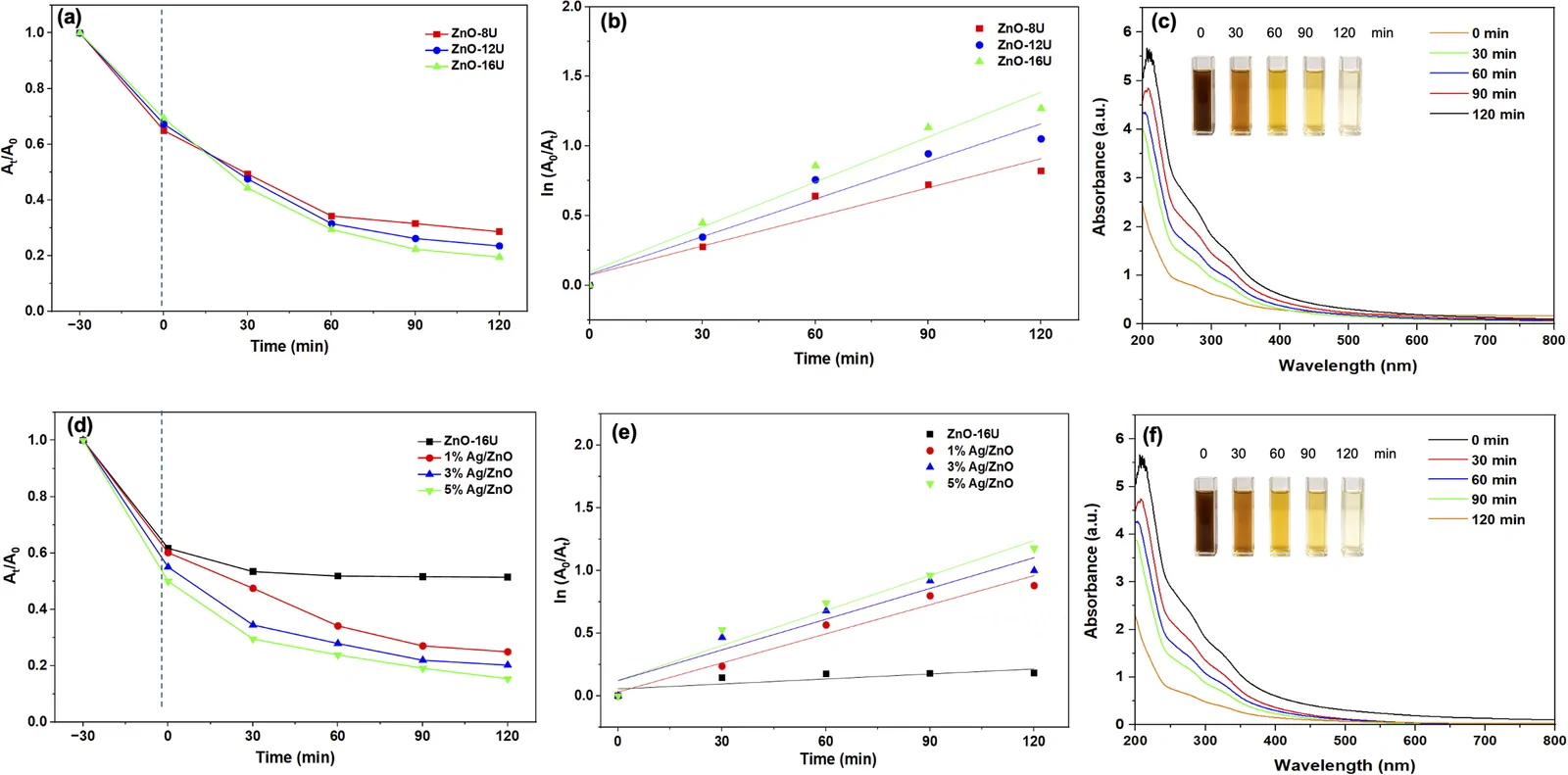
Photocatalytic decolorization of POME over ZnO and Ag/ZnO coral-like nanoplates. (a) POME decolorization profiles under UV irradiation over ZnO-8U, ZnO-12U, and ZnO-16U, (b) corresponding pseudo-first-order kinetic plots, and (c) UV-vis spectral evolution of POME over ZnO-16U. (d) POME decolorization profiles under visible-light irradiation over ZnO-16U and Ag-modified ZnO-16U samples with different Ag loadings of 1, 3, and 5 wt%, (e) corresponding pseudo-first-order kinetics plots; and (f) UV-vis spectral evolution of POME over 5 wt% Ag/ZnO. Insets show the corresponding color changes during photocatalytic treatment.

To further evaluate organic-load reduction and obtain quantitative evidence of partial mineralization in the wastewater matrix, COD and TOC removal efficiencies were measured for post-treated POME after 120 min of visible-light irradiation. As shown in Table 2, pristine ZnO-16U achieved 50.0% COD removal and 48.0% TOC removal, indicating moderate reduction of oxidizable and carbon-containing organic constituents in the complex POME matrix. In contrast, 5 wt% Ag/ZnO exhibited markedly higher COD and TOC removal efficiencies of 83.0% and 82.0%, respectively. These results are consistent with the UV-vis decolorization results and confirm that the enhanced performance of Ag/ZnO is not limited to color reduction, but also contributes to substantial organic-load reduction in post-treated POME. The improved COD and TOC removal can be attributed to enhanced visible-light absorption, suppressed electron–hole recombination, and more efficient generation of reactive oxygen species at the Ag/ZnO interface. Although complete mineralization was not achieved within 120 min, the high COD and TOC removal efficiencies demonstrate the strong potential of 5 wt% Ag/ZnO for visible-light-driven treatment of post-treated POME.

**Table 2:** COD and TOC removal efficiencies after 120 min of visible-light photocatalytic treatment of post-treated POME using ZnO-16U and 5 wt% Ag/ZnO

Sample	Photocatalyst	Light source	Irradiation time	COD removal (%)	TOC removal (%)
Post-treated POME	ZnO-16U	Visible light	120 min	50.0	48.0
Post-treated POME	5 wt% Ag/ZnO	Visible light	120 min	83.0	82.0

The reusability performance of ZnO-16U and 5 wt% Ag/ZnO photocatalysts during the degradation of MO and decolorization of post-treated POME over five consecutive photocatalytic cycles is shown in Fig. 8(a). Both catalysts retained a high level of activity throughout the repeated experiments, indicating good operational stability and resistance to deactivation under irradiation conditions. For MO degradation, ZnO-16U exhibited a gradual decrease in degradation efficiency from approximately 90% in the first cycle to 85% after the fifth cycle, corresponding to an activity retention of about 94%. In contrast, 5 wt% Ag/ZnO maintained a higher photocatalytic performance, with the degradation efficiency decreasing slightly from approximately 92% to 88%, corresponding to a retention of about 96%. The superior stability of Ag/ZnO suggests that Ag incorporation not only enhances photocatalytic activity but also contributes to improved catalyst durability during repeated operation. The smaller decrease in efficiency for Ag/ZnO suggests stronger resistance to photocorrosion compared with bare ZnO.^17,24,59^ A similar trend was observed for POME treatment by 5 wt% Ag/ZnO. Although the decolorization efficiency gradually decreased from approximately 85% to 79% after five cycles, the catalyst retained more than 90% of its initial activity. The slightly greater loss of performance compared with MO degradation is expected because POME contains a complex mixture of suspended solids, phenolic compounds, lignin derivatives, and other recalcitrant organic species. During repeated operation, these constituents may adsorb onto the catalyst surface, partially block active sites, or hinder light penetration, resulting in a gradual decline in photocatalytic efficiency.^11,12,25,31^ Despite the challenging wastewater matrix, the Ag/ZnO catalyst maintained nearly 80% decolorization efficiency after five consecutive cycles, demonstrating good long-term stability under realistic treatment conditions. This result highlights the practical applicability of the catalyst for wastewater remediation, where resistance to fouling and sustained activity are critical performance requirements. Similar improvements in catalyst durability following Ag incorporation have been reported for Ag/ZnO systems used in dye degradation and wastewater treatment, where the metal–semiconductor interface contributes to both enhanced photocatalytic activity and improved resistance to photocorrosion.^22–24,27,60^ To further assess the operational stability and extent of metal release of the 5 wt% Ag/ZnO catalyst, Ag and Zn leaching after the fifth photocatalytic cycle was analyzed, and the results are provided in Table S1. Zn leaching was 0.12 mg L^−1^ for MO and 0.23 mg L^−1^ for POME, corresponding to leaching percentages of only 0.0152% and 0.0301%, respectively. Ag leaching was lower, with dissolved Ag concentrations of 0.0035 mg L^−1^ for MO and 0.0038 mg L^−1^ for POME, corresponding to 0.0070% and 0.0076% leaching, respectively. The low concentrations of dissolved Ag and Zn after repeated use indicate limited metal release from the catalyst, supporting the good operational stability of the Ag/ZnO heterostructure during photocatalytic treatment.

**Fig. 8:**
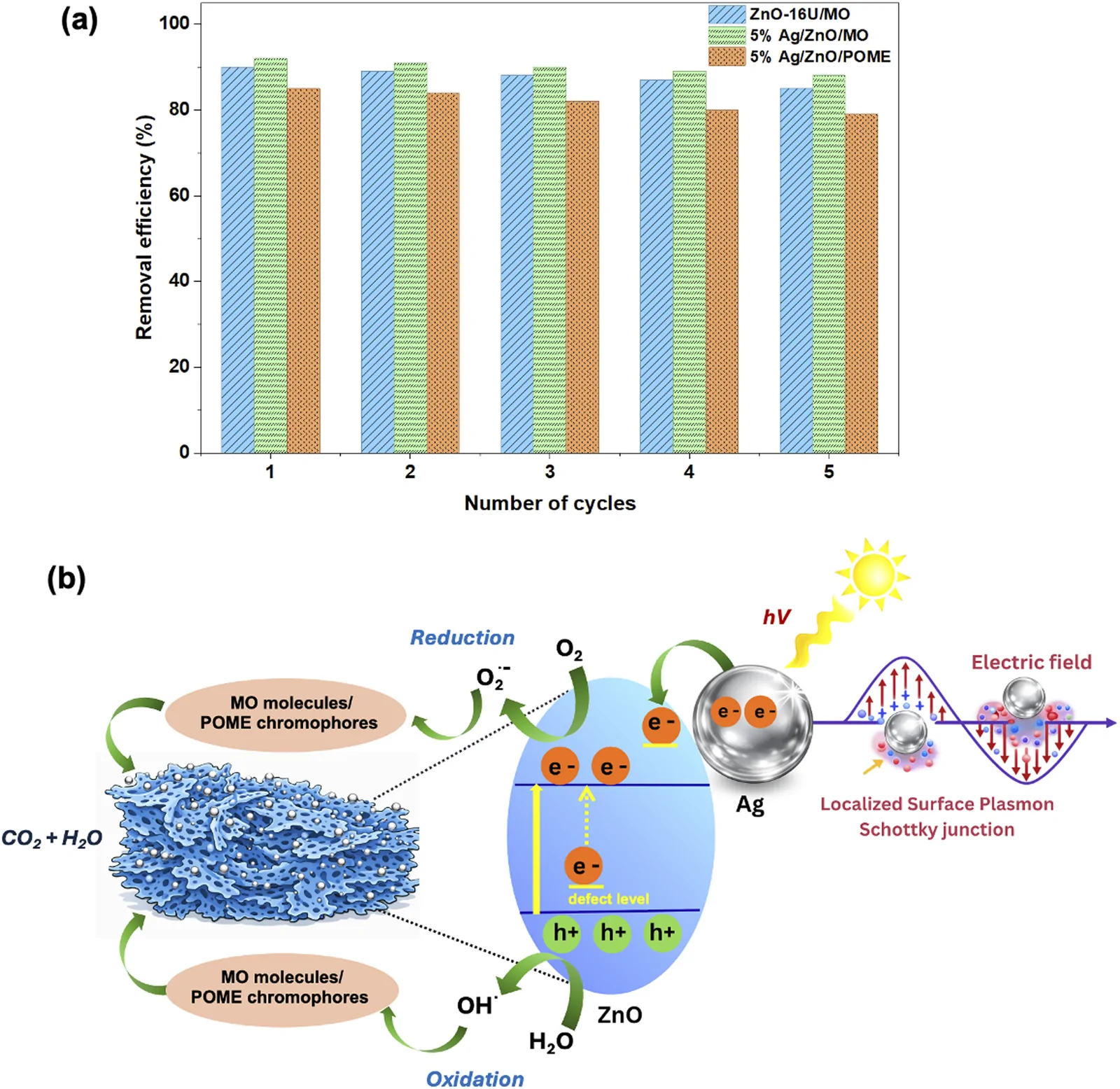
(a) Cycling stability and reusability of ZnO-16U and 5 wt% Ag/ZnO photocatalysts during the photocatalytic degradation of MO and decolorization of POME over five successive reaction cycles. (b) Proposed photocatalytic mechanism of the Ag/ZnO coral-like nanoplates under visible-light irradiation.

The proposed photocatalytic mechanism of the Ag/ZnO heterostructure under visible-light irradiation is illustrated in Fig. 8(b). Upon illumination, the localized surface plasmon resonance (LSPR) effect of Ag nanoparticles enhances visible-light absorption and generates energetic electrons at the metal surface. Simultaneously, the Ag/ZnO interface forms a Schottky junction that facilitates directional electron transfer and suppresses electron–hole recombination.^22,23^ The separated electrons participate in the reduction of dissolved oxygen to produce superoxide radicals (˙O_2_^−^), while photogenerated holes oxidize surface hydroxyl groups or water molecules to generate hydroxyl radicals (˙OH). These reactive oxygen species subsequently attack and decompose organic pollutants into smaller intermediates and may ultimately mineralize them into CO_2_ and H_2_O.^51,52^ The synergistic combination of plasmon-enhanced light harvesting and efficient charge separation accounts for the superior photocatalytic activity and stability of the Ag/ZnO system.

## 4 Conclusions

Porous coral-like ZnO nanoplates were successfully synthesized by a urea-assisted hydrothermal method and modified with Ag to develop a visible-light-responsive photocatalyst for both model dye degradation and real post-treated POME treatment. Among the pristine samples, ZnO-16U exhibited the highest photocatalytic activity despite its lower BET surface area, indicating that photocatalytic performance depended on the combined effects of morphology, structural accessibility, defect-related optical properties, and charge-transfer behavior rather than surface area alone. Under UV irradiation, ZnO-16U achieved approximately 90% MO degradation within 120 min. Ag modification markedly enhanced visible-light activity while preserving the coral-like ZnO structure. The 5 wt% Ag/ZnO sample showed the best performance, achieving approximately 92% MO degradation and 85% POME decolorization after 120 min. In addition, treatment of post-treated POME resulted in 83% COD removal and 82% TOC removal, demonstrating that the photocatalytic effect extended beyond color removal to substantial reduction of the residual organic load. The enhanced activity is attributed to improved visible-light harvesting through Ag-induced LSPR together with more efficient interfacial charge separation at the Ag/ZnO heterostructure. The 5 wt% Ag/ZnO catalyst also maintained approximately 79% POME decolorization after five reuse cycles, while the very low release of Ag and Zn indicated good material stability during repeated operation. Overall, the results demonstrate that integrating morphology engineering with Ag modification is an effective strategy for improving the visible-light photocatalytic performance and operational stability of ZnO. The developed Ag/ZnO coral-like nanoplates show potential for the treatment of dye-contaminated water and post-treated POME from palm oil milling operations.

## Author contributions

Conceptualization, P. P., and S. T.; methodology, P. P., and S. T.; validation, P. P., T. B., M. L., and S. T.; formal analysis, P. P., T. B., M. L., and S. T.; investigation, P. P., T. B., S. Y., P. B., and S. T.; resources, P. P., T. B., and S. T.; visualization, P. P., and S. T.; writing – original draft preparation, P. P., and S. T.; writing—review and editing, P. P., T. B., M. L., and S. T.; and supervision and funding acquisition, S. T. All authors have read and agreed to the published version of the manuscript.

## Conflicts of interest

There are no conflicts to declare.

## Supplementary Material

RA-016-D6RA06010B-s001

## Data Availability

The data supporting this article have been included within the manuscript and its supplementary information (SI). Supplementary information: additional raw and processed data, including UV-vis absorbance spectra, photocatalytic degradation and decolorization profiles, kinetic analysis data, operational-parameter screening data, photoluminescence spectra, physicochemical characterization data, COD and TOC measurements, metal-leaching results, and catalyst reusability data, are available from the corresponding author upon reasonable request. See DOI: https://doi.org/10.1039/d6ra06010b.
