# Supplementary material for: Morphology-engineered Ag/ZnO coral-like nanoplates for visible-light photocatalytic degradation of methyl orange and decolorization of palm oil mill effluent
Source: RSC Adv. 2026 Oct 30;16(49):67257–72. doi: 10.1039/d6ra06010b (PMC13720777; doi:10.1039/d6ra06010b)
Supplement: Supplementary file 1 [file RA-016-D6RA06010B-s001.pdf]

Supplementary Information (SI) for RSC Advances.
This journal is © The Royal Society of Chemistry

ELECTRONIC SUPPORTING INFORMATION

Morphology-engineered Ag/ZnO coral-like nanoplates for visible-light photocatalytic degradation of methyl orange and decolorization of palm oil mill effluent

Piangjai Peerakiatkhajohn[a], Teera Butburee[b], Miaoqiang Lyu[c], Salwatee Yu-soh[a], Padsiriporn Budwang[a], and Supphasin Thaweesak[d*]

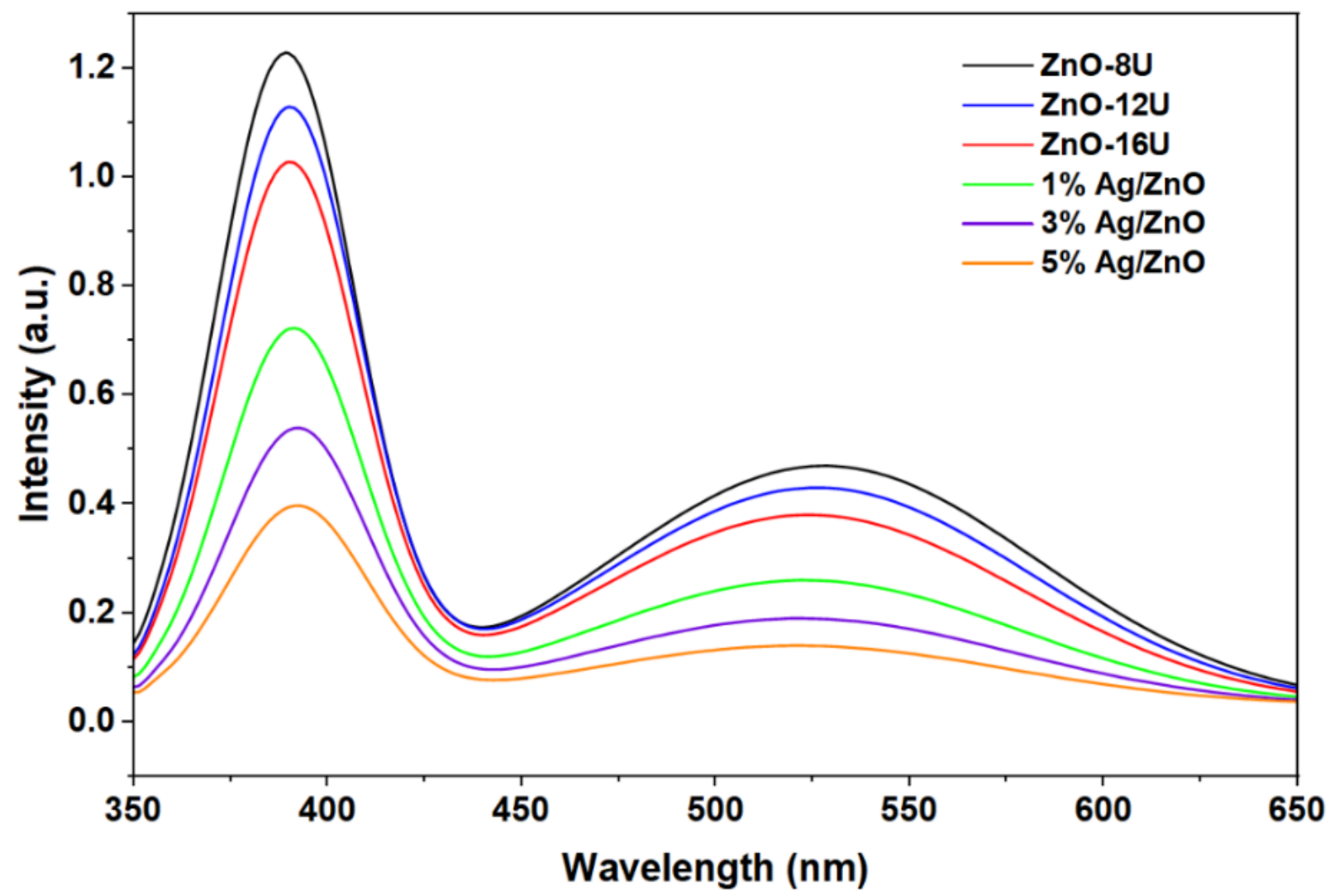

Fig S1. Photoluminescence spectra of ZnO and Ag-modified ZnO samples with different Ag loadings.

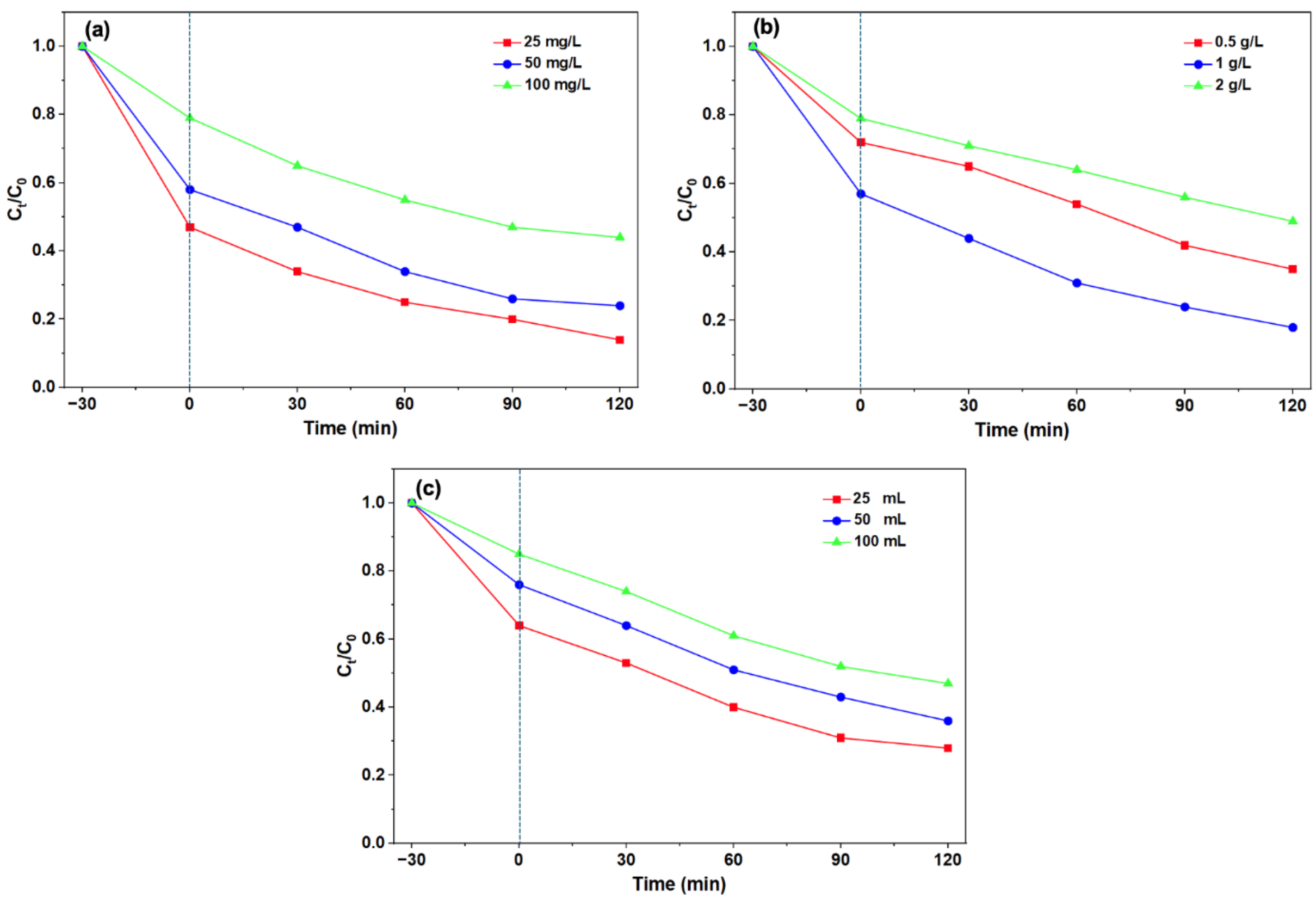

Fig S2. Effects of operating parameters on the photocatalytic degradation of methyl orange (MO) using ZnO-16U under UV-light irradiation: (a) initial MO concentration, (b) photocatalyst dosage, and (c) reaction volume under a fixed catalyst mass

Table S1. Dissolved Zn and Ag concentrations and corresponding leaching percentages from 5 wt.% Ag/ZnO after photocatalytic treatment of MO and post-treated POME.

Treated solution	Metal	ICP-OES concentration (mg L⁻^{1})	Leaching (%)
MO	Zn	0.12	0.0152
MO	Ag	0.0035	0.0070
POME	Zn	0.23	0.0301
POME	Ag	0.0038	0.0076

Leaching percentage was calculated based on the amount of dissolved metal detected in the filtrate after the fifth photocatalytic cycle relative to the initial amount of the corresponding metal present in the catalyst. The catalyst dosage was 1.0 g L⁻^{1}, and the reaction volume was 50 mL.
